# Locating Disability Within a Health Justice Framework

**DOI:** 10.1017/jme.2023.6

**Published:** 2022

**Authors:** Jasmine E. Harris

**Affiliations:** 1: UNIVERSITY OF PENNSYLVANIA CAREY LAW SCHOOL, PHILADELPHIA, PA, USA.

**Keywords:** Disability Law, Disability Justice, Health Justice, Health Law, Intersectionality

## Abstract

This Article explores the connections between disability and health justice in service of further tethering the two theories and practices. The author contends that disability should shift from marker of health inequity alone to critical demographic in the analytical and practical application of health justice. This theoretical move creates a more robust understanding of the harms of health injustice, its complexities, and, remedially, reveals underexplored legal and policy pathways to promote health justice.

According to Dr. William Kissick, three core principles — access, cost, and quality — tradeoff to shape the contours of healthcare law and policy.^1^ Concerns with these tradeoffs have occupied health law and policy for decades. Health justice scholars have moved the needle beyond the “iron triangle” to recognize that this understanding of health law “lacks the ambition and scope” to respond to difficult cross-cutting structural issues.^2^ Part of their efforts to expand the relevant lens of research and scholarly inquiry has focused on the causes of inequitable healthcare outcomes by attending to the social determinants of health.^3^ The rhetorical, analytical, and practical positioning of disability within a health justice framework affects problem identification, an understanding of the harms, and remedial efforts, but also sends a clear message to disabled people about the value of disability as identity and their quality of life.

This article contributes to ongoing conversations about the relationship between disability^4^ and health justice, identifies points of overlap, tension, and departure through a case study, and calls for greater integration of disability into health justice scholarship and practice.^5^ For purposes of this discussion, disability refers to a physical or mental impairment that limits an individual’s functional capacity while also recognizing the “disabling” nature of the impairment can be rooted in and exacerbated by structural and institutional choices that fail to account for nonnormative bodies and minds.^6^ The relationship of disability to health and health law is complex and the subject of scholarly debate due to narrow conceptions of disability as an individual health outcome rather than a socio-political identity.^7^ Is disability a medical impairment, a diagnosis, a “bad difference” or a “mere difference” on a broad spectrum of human capability? These debates are outside of the scope of this Article;^8^ however, the very existence of these debates affects whether and where disability appears in health justice conversations.

This article contributes to ongoing conversations about the relationship between disability and health justice, identifies points of overlap, tension, and departure through a case study, and calls for greater integration of disability into health justice scholarship and practice.

This article calls attention to disability as a demographic and health outcome and argues that both are necessary to build a sustainable bridge between disability and health justice. Disability is more than just a downstream data point that evinces inequities along other axes of marginalization such as a race, gender, class, sexuality, immigration, or nationality status. Disability should be a meaningful part of upstream legal and policy interventions to make good on the theory and practice of health justice. Reduction of disability to health outcomes reinforces the medical model of disability — a limited view of disability as individual, physiological deficit — that the disability rights movement has worked to change.^9^ Disabled people encounter barriers to healthcare access, and research suggests that when they access healthcare institutions, they may experience poorer outcomes.^10^ But access barriers and poor outcomes are only part of the story.

Attention to the complexity of disability as socio-political identity and as health status positions disability as a critical analytic point of entry into a discussion of social inequities that, at times, uses disability as a label or tool of structural subordination and social sorting. Asking the “disability” question, as Professor Mari Matsuda has explained, in the context of other marginalized identities, can surface cross-movement opportunities for liberation.^11^ Thus, examining where disability is situated in the health justice agenda opens a discussion about disability as identity and disability as evidence of health injustice.^12^ Moreover, it continues the work of scholars concerned with how to reconcile antidiscrimination law’s post hoc interventions and health law’s prophylactic dimensions.^13^ This article also contributes to conversations among scholars and practitioners calling for greater deployment of intersectionality in the context of race, disability, and health justice.^14^

This article proceeds in three parts. Part I describes the origins of disability and health justice as movements and analytical frames operating on parallel tracks and the recent work seeking to engage both. Part II offers a fictionalized case study of “Aida” based on real cases to illustrate what a disability lens adds to the health justice framework.^15^ Part III repositions disability as a demographic in the health justice framework to advance health equity and, in doing so, advances disability law and policy as potential health justice remedies.

## I. Disability and Health Justice

Disability justice and health justice offer two critical lenses to better understand structural subordination. Both critical approaches are deliberately reactive, relatively recent and fluid. Disability justice responds to a focus on disability *rights*. Emphasis on rights draws attention to individual harm without the peripheral context. It also privileges the remedial role of law, and thus, relies heavily on lawyers to construct narratives of legal harm that respond to rigid legal categories and remedies rather than lived experiences.^16^ A rights-based approach to justice emerged during the civil rights movement to offer individual rights of private action for discriminatory conduct perpetrated by state and private actors. It privileges courts and legal actors as arbiters of justice. The disability rights movement followed a preexisting civil rights playbook by privileging individual rights and remedies to address discrimination. These civil rights movements sought seeking equitable treatment and full social, economic, and political citizenship for marginalized communities. Disabled people of color and queer and trans people sought to shift the focus on post-hoc legal interventions to institutional and systemic discrimination that discriminated *by design* and de-centered the most marginalized within the disability community.^17^ Disability justice rejects the single issue, undifferentiated focus that fails to center the non-White experiences and those of people with non-mobility impairments.^18^ It targets ableism as the root of disability subordination and advances ten principles of justice: intersectionality; leadership by those most impacted; anti-capitalist politics; cross-movement solidarity; recognizing wholeness; sustainability; commitment to cross-disability solidarity; interdependence; collective access; and collective liberation.^19^

Similar to disability justice, health justice responds to a narrower vision of health law focused on individual access to healthcare, the cost, and quality of that care. Health justice is a theoretical framework that deploys law and policy to eliminate racial and socioeconomic health disparities.^20^ “The framework centers on engaging, elevating, and increasing the power of historically marginalized populations to address structural and systemic barriers to health, as well as to compel the adoption of rights, protections, and supports necessary to the achievement of health justice.”^21^ Although its contours are rapidly changing in response to greater theorizing and practice, it is primarily concerned with the social determinants of health and how they create or exacerbate racial and socio-economic health disparities.^22^ Professor Angela Harris and Aysha Pamukcu argue that rather than biological vulnerability, marginalized populations in health literature are vulnerable because of social and political barriers to achieving health equity.^23^ Health justice scholars recognize that law itself is a key social determinant that affects health equity and seek to reform law and policy to create a “health in all policies” agenda.^24^ This praxis shifts law from a strictly remedial role to a prophylactic one.

Until relatively recently, disability and health law have operated on parallel tracks. Scholars such as Professor Jessica Roberts have probed the interconnectivity of disability and health law arguing that health law should be viewed as a form of disability law because of its practical impact on health disparities experienced by disabled people.^25^ That is, the substantive schism between civil rights and health law functionally impedes progress, further supporting a proposition advanced by Professor Sam Bagenstos that the “future of disability law” lies in public benefits law (in which he includes Medicaid and access to health supports).^26^

More recent work by disability law scholars touts the utility of a health justice framework to advance principles of disability justice.^27^ Professor Katherine Macfarlane has applied a health justice framework to the process of reasonable accommodations.^28^ Macfarlane argues that Title I of the Americans with Disabilities Act does not require formal documentation to request a reasonable accommodation in the context of employment; rather, this practice reflects a customary norm rooted in suspicion about fraud and abuse.^29^ A health justice approach, Macfarlane notes, would honor self-claims of disability without subjecting disabled people to a costly process that centers medical expertise rather than human dignity.^30^ Professor Robyn Powell invokes a health justice framework to understand the impact of Covid-19 on disabled people and through the Covid-19 example, argues for inclusion of disabled people within the health justice framework.^31^ Powell connects the increased vulnerability of disabled people to Covid-19 to core social determinants of health such as lower education levels and employment rates with high rates of poverty and food insecurity.^32^ In this way, public discourse about “vulnerability” to Covid-19 moves from “preexisting” or “underlying” health conditions as individual deficits to the structural conditions that create precarity. Furthermore, Professors Elizabeth Pendo and Kelly Dineen write at the intersection of disability and health law. Their most recent work seeks to advance a health justice approach to substance use disorder (SUD) in three ways. First, they identify the structural barriers to health equity for people with SUD noting the disproportionate impact of SUD on people of color. Second, by surfacing the particular experiences of people of color with SUD in health care, they argue that an intersectional approach to enforcement grounded in disability rights laws could ameliorate existing inequities.^33^

Analytically, the health justice framework complements disability rights and disability justice frameworks by focusing remedial interventions on communities and environments and centering affected individuals in the diagnostic and remedial processes. But there are multiple points of disability injustice along the health justice pipeline. One critique of health law’s treatment of disability (at least historically) is the positioning of disability as a health outcome and not a distinct demographic axis of vulnerability to be considered with and alongside other identities.

When disability operates as a negative health outcome alone, it reinforces disability as a “bad difference” and an “impairment” through a medicalized lens. This works at cross-purposes with the disability justice movement making it more difficult to position disability as “a different difference” or a socio-political identity.^34^ This may be especially true for people at the intersection of multiple marginalized identities who may not wish to claim “disability” when it is understood as a deficit or impairment.^35^ This is not to say that disability cannot (or should not) be used as evidence of structural subordination; rather, the danger exists when discourse around disability is limited to disability as the product of inequitable systems without also situating disability as an identity and demographic, one that also disproportionately experiences health inequities. Recent work by Professor Rabia Belt offers the “fat prisoners’ dilemma” as an example of the tension between efforts to reduce the social determinants of health in the context of improving the structural conditions of incarceration while also recognizing that doing so will necessarily reduce the number of disabled people and may position the existence of disability itself as a harm to be remedied.^36^ Belt argues that attention to intersectionality alone will not remedy the situation nor address this tension and, instead, calls upon legal scholars to broaden the analysis and investigate “how injustice produces impairment, which in turn creates people who are multiply marginalized.^37^ Similarly, this article illustrates why attention to disability as either demographic or health outcome alone is insufficient to advance health justice. The role of disability in these discussions is complex and nuanced. Disability can be a health outcome, the result of structural subordination because of, for example, racism, environmental harm, and/or ableism. In this sense, it is both a sign of structural inequities as well as the product of that inequity which, as Professor Belt contends, may make disability a remedial target. This can complicate “claiming disability” as an identity at an individual level^38^ and attempts to “take disability public”^39^ as a means of widespread normative shifts about disability in society.

The next section demonstrates the problem of positioning disability downstream as a health outcome without attending to disability’s upstream analytical value as a preexisting identity. It illustrates the intersections between disability and health justice and highlights areas missed for research and advocacy when disability is not an active lens used to evaluate the harm and conceptualize the remedies available. Instead, disability becomes a way to understand and measure harms based on other identity categories such as race, gender, socio-economic status, or sexuality, rather than, at least in part, inequitable outcomes compounded by status as a disabled person. The facts presented represent a composite case study and have value to better understand why framing (and asking the right questions) matters in the context of disability and health justice.

## II. Case Study: “AIDA”

Consider the following case study of Aida, a disabled person with a developmental disability (cerebral palsy) and episodic chronic illness. The facts that follow derive from a typical set of questions asked during health screenings, interviews, or legal intakes with the goal of defining a client’s problem and evaluating potential interventions and supports, including, in the legal context, whether to take a case. How does the health justice pipeline operate for her? While there is no set path into this analysis, two analytical starting points are through employment and healthcare. Aida, a Puerto Rican woman, lives in New York City in the South Bronx in public housing with her partner and Aida’s seventeen-year-old son, Marco, who has asthma and, like his mother, has a developmental disability.^42^ Aida works between five and fifteen hours a week in Manhattan as a contract worker for a small mom and pop catering business.^43^ Aida receives federal income support through the Supplemental Security Income (SSI) program for her and her son and access to the Supplemental Nutrition Assistance Program (SNAP) (formerly known as the food stamps program). The catering company does not offer private health insurance, but Aida receives Medicaid managed by New York state. Aida has no savings and lives month to month.

One might conclude from this information that Aida lives below the poverty line. Application of the principles of Kissick’s iron triangle, may suggest that Aida needs lower cost access to better quality health insurance. The entry point here would be an analysis of Aida’s individual circumstances and identification of the costs of care and other barriers to her access that trade off against each other. A health justice approach might suggest that her disabilities demonstrate inequitable access to gainful employment and wealth accumulation and may seek additional information based on her disability as it related to the institutions she encounters. Application of a disability lens centering her identity as a Latina with a disability offers additional information and insights. Congress created the SSI program, for instance, to address poverty associated with disability but operationalized this program based on an assumption that disability was incompatible with employment.^44^ The legal definitions of “income” and its permissible exemptions may present disabled people with a difficult choice between seeking sustainable employment or accepting less stable employment or less compensation to ensure continued receipt of benefits, most often, to protect access to Medicaid coverage.^45^ SSI’s “marriage penalty” is another example of law’s constraints on disabled people’s choices.^46^ SSI recipients may lose their SSI benefit and Medicaid if they marry a person with a higher income or overall assets by virtue of program design. The Social Security Act (SSA) includes a spouse’s income and assets in its overall eligibility determination; where two recipients of SSI decide to marry, the SSA may not terminate benefits entirely but both spouses would receive a twenty-five percent reduction of monthly benefits, including any additional benefits for Aida’s son if he also receives SSI benefits (which he does).^47^

Immigration/nationality status offers another important analytical entry point typical of a health justice framework.^48^ Positioning disability downstream might miss an important structural barrier to health justice that turns on Aida’s identity as a disabled Puerto Rican. Although Aida’s permanent residence is in New York, she spent four years in Puerto Rico caring for her dying father. Puerto Ricans are U.S. citizens; however, pursuant to the Jones Act and the Supreme Court’s decisions in the insular cases, Puerto Rico’s territorial status constrains certain rights, responsibilities, and entitlements of citizenship for those living in Puerto Rico rather than the continental United States. This includes receipt of SSI benefits.^49^ Congress’s decision to exclude residents of Puerto Rico from eligibility for the SSI safety net program meant that Aida’s change in address during that four year period made her ineligible for receipt of SSI benefits.^50^ Aida, unaware of this, continued to use her benefits during those four years but recently, now living in New York again, received a letter from the Social Security Administration seeking to recoup more than twenty thousand dollars for payments while she lived in Puerto Rico or potentially face additional monetary and even potential criminal charges for “fraud.”^51^

Housing offers another analytical path to understand disability within the health justice framework. Disability as demographic enhances an understanding of Aida’s housing precarity, another typical element of a health justice framework. Aida and her family reside in public housing regulated by the New York City Housing Authority (NYCHA), the largest public housing administration in the country.^52^ Aida has lived in the same NYCHA unit since she was a child and, despite years of open work orders seeking to address heating and plumbing issues, Aida could not secure affordable housing elsewhere in New York City and the waitlists for other subsidized housing, including for other units in the same public housing development, were years long for similar conditions.^53^ Aida has chronic obstructive pulmonary disease (COPD) in addition to cerebral palsy, the former which has been exacerbated by mold from a leaking pipe. A recent study reported that more than eighty percent of NYCHA buildings had mold and other conditions that could contribute to asthma in children, one of the leading causes of school absence. In 2008, for example, school-aged children missed more than 10.5 million school days, with children from low-income communities among those most affected by school absences.^54^

A health justice analysis that tracks disability (asthma and COPD as a product of or corollary to mold exposure) solely as a health outcome for vulnerable populations along the axis of race, fails to capture the compounded discrimination Aida experiences as a *disabled* Latina. Ensuring access to healthcare, reducing cost, or improving its quality will not resolve health inequities for Aida or others who are similarly situated.^55^ Moreover, Aida’s disabilities cannot be reduced to her environment; for example, Aida’s cerebral palsy, while congenital, has actual functional impairments that cause her pain. To reduce her disability to a social construction alone ignores the lived reality of physical pain.

Finally, health justice interrogates law as a source of health inequities. Situating Aida as a disabled person, the question becomes “how do law and policies create or reinforce health inequities for disabled people?” Aida’s case study demonstrates the ways in which laws and policies designed by and for able-bodied and neurotypical individuals have created an inaccessible world where disabled people must ask able-bodied and neurotypical individuals and institutions to accommodate them, individually, without expecting the fundamental redesigns that change the baseline norms themselves. The following part argues that shifting disability in a health justice framework offers a descriptively richer and more complete account of health inequities and opens remedial avenues available when disability is understood as demographic in addition to evidence of health inequity.

## III. Reorienting Disability in the Health Justice Framework

Reorienting disability in the health justice framework allows us to see additional systemic barriers to health that complement disability antidiscrimination laws. Professor Sam Bagenstos argued that the future of disability law lies in public benefits law; this argument complements the health justice model.^56^ His argument is that if a disabled person does not have access to social supports, for example, personal assistance, transportation, and accessible housing, the individual cannot get to the job site and so disability rights against employment discrimination is not meaningful because they have no job to get to. Or, if they are employed but do not have affordable, safe housing or transportation, they will have greater difficulty maintaining the job in the future. Once in the workplace, the disabled person may request reasonable accommodations to assist them in performing their job responsibilities. When the systems work in tandem, the individual is employed, their disability accommodated, and they can live in a community setting and participate in social, economic, and political life.^57^

Repositioning disability uncovers additional social determinants or adds dimension to existing focal points in health justice. First, a health justice lens connects the leaking pipe and related environmental hazards in Aida’s apartment in New York City’s Housing with asthma and perhaps Marco or Aida’s developmental disabilities. Professor Emily Benfer proposes a health justice intervention to the NYCHA environmental harms would be to shore up federal agency oversight and for Congress to increase available funding to ensure compliance at the local level.^58^ Media reports suggest that agency regulations and guidelines allowed inspectors in Washington, DC to visually assess properties for evidence of mold or lead, a practice which created space for some landlords to simply paint over the problem without detection.^59^

But shifting disability to demographic may identify additional systemic barriers to health equity related to housing. When Aida’s father passed, the NYCHA requested that Aida sign the lease as the head of household. Because her father listed her previously as a person with a developmental disability, and because she and Marco receive services from the state Office for People with Developmental Disability, NYCHA would not let Aida sign the lease and, instead, requested that she show an order from a court saying she had the legal capacity to enter into a contract or that she ask someone else sign the lease.^60^ The NYCHA form included language about the inability to sign “because of a physical or mental disability” without qualifying language such that the NYCHA office read this as a categorial exclusion. Because Aida did not want to lose her housing, she asked her partner to sign the lease. Aida is listed as an occupant along with her son on the lease but not the primary occupant and head of household meaning that she may have fewer formal rights with respect to holding NYCHA accountable for the conditions in her apartment that may have contributed to or exacerbated Aida’s and Marco’s disabilities.

In addition, her son, Marco is about to turn eighteen years old and wants to live independently. Both Aida and Marco receive services from the state Office for People with Developmental Disabilities (OPDD). The OPDD serves as a clearinghouse for services and supports for people with developmental disabilities. Marco wants to live in a group home and work in the community. The Medicaid Home and Community Based Services (HCBS) Waiver Program provides funding for Marco to live in the community and avoid larger institutional settings like nursing homes and the largescale institutions of the past.^61^ However, due to staffing shortages, the group home OPDD secured for Marco will close its doors, leaving not only Marco without a community-based housing option but also leaving seven current residents with intellectual and developmental disabilities without housing. While the closure of the group home may appear to be a limited occurrence or even related to the pandemic’s general employment crisis of workers, the crisis of care workers and long-term care are preexisting systemic issues exacerbated by the pandemic.^62^ Lack of funding to pay care workers and maintain operating budgets has led group home operators across the country to close their doors, or, in some states like Oklahoma, to have residential openings but no staff to operate them.^63^ In 1999, the Supreme Court of the United States held in *Olmstead v. L.C.* that unnecessary institutionalization of the sort Marco and the seven prior residents of the group home face when they are willing to and capable of living in community settings amounts to disability discrimination under Title II of the Americans with Disabilities Act.^64^ In this respect, therefore, identifying Aida and Marco as people with disabilities highlights additional systemic barriers to health equity and also offers a potential legal remedy through the Americans with Disabilities Act.

Second, and relatedly, the question of legal capacity for people with intellectual and developmental disabilities arises not only in the context of the power to enter into a contract (such as signing a lease) but also in the context of other decisions such as education and health care where customary norms of guardianship and substitute decision-making track informed consent’s treatment as the gold standard for decisional agency. If Marco requires some support in decision-making, the systems with which he interacts — education and health care — may call into question his legal capacity. For example, when Marco turns eighteen, under special education law, he reaches the age of majority and his educational rights transfer from Aida to him. During his transitional individualized education program (IEP) meeting, the school district can shape Marco’s future decisional agency. They might advise Aida that she ought to seek a guardianship order over her son (assuming that a probate judge would agree that she herself has the capacity to act on his behalf) to ensure that she can stay involved in his educational and health care decisions. If Aida takes this path, she will effectively strip decisional capacity from Marco including potentially depending on the state of residence his right to serve on a jury or to vote. Alternatively, the school district can advise Aida that, although Marco will hold the right to make decisions about his education and health and other legal matters when he turns eighteen, assuming Marco agrees, she can remain involved informally or formally through supported decision-making.^65^ Some states such as Texas have formalized supported-decision-making to allow for broader acceptance given the strong cultural norms and practices around requiring formal court interventions and monitoring of the decisions of people with mental disabilities.^66^ As the public learned recently through the case of Britney Spears, guardianship (or conservatorship as it is known in some states) can amount to civil death.^67^ Furthermore, once a guardianship order is issued, it can be extremely difficult to undo, in part, because, in some states, the evidentiary standard of proof to modify or terminate an order of guardianship is “clear and convincing” evidence, a higher standard than the default in civil cases of “preponderance of the evidence.”^68^

Third, consideration of disability as demographic or identity highlights constraints on wealth accumulation for people with disabilities, in particular. Adults with disabilities living in community settings have a poverty rate of more than twice that of nondisabled adults: 25.9% compared to the poverty rate of nondisabled adults of 11.4%.^69^ High poverty rates relate to high rates of unemployment and underemployment^70^ among people with disabilities like Aida who must also balance the desire to work and the availability of gainful employment with strict means tests for receipt of vital health insurance and supplemental income supports. Added to these sobering statistics, the costs of living with disability in society are significantly greater and range from financial expenditures to time spent on the administrative tasks associated with living with disability, what some refer to as the “disability tax” or what Professor Elizabeth Emens calls “disability admin.” ^71^

Health justice scholars cognizant of Aida’s and Marco’s disabilities and the structural barriers to wealth accumulation rooted in their identities as disabled recipients of certain public benefits might identify the potential legal and policy reforms designed to allow disabled people to shelter certain income from public benefits means tests. For example, the Achieving a Better Life Experience (ABLE) Act signed into law in 2014 allows qualified individuals with disabilities to set up tax free savings accounts exempt from consideration in means test eligibility for Social Security and Medicaid.^72^ These accounts are modeled after the 529 college savings accounts to advance a preferred policy goal — save for college tuition or, in the case of ABLE, to offer an additional source of financial assets for people with disabilities. ABLE accounts deploy tax law to create an opening for limited wealth accumulation. However, only those who were diagnosed before age 26 are eligible (proof of disability required), savings must not exceed $100,000 to remain eligible for public benefits and the money can be withdrawn tax free only to pay for a list of enumerated “qualified disability expenses” such as transportation, education, healthcare, death expenses, or legal fees. In addition, each state implements its own ABLE program with no standardized terms or data collection across states; such decentralization is typical with benefits programs related to disability. An example of how the ABLE Act may create greater health inequities is the Medicaid Estate Recovery Recapture provisions which require any money remaining in ABLE accounts, upon the death of the account holder, to return to the federal government who acts as first creditor. This means that should Aida establish an ABLE account and then pass away, Marco cannot access the balance of money in the account. Thus, the notion of wealth accumulation is temporal and cannot pass to children. Aida, instead, can create a separate ABLE account for Marco’s benefit subject to the same limitations.

Thus, considering disability at the onset as an interdependent variable and demographic allows for a broader view of the interconnected systems that produce health injustice for disabled people like Aida and Marco. At the same time, it adds additional legal and policy remedies to the overall health justice toolbox. Disability expands the universe of downstream, post hoc legal remedies not from a disability as damages/tort perspective, but rather from an antidiscrimination, perspective. Federal disability laws such as the Americans with Disabilities Act, Section 504 of the Rehabilitation Act of 1973, the Affordable Care Act, Individuals with Disabilities Education Act, and the Fair Housing Act, in addition to state analogs, offer Aida and similarly situated individuals the deterrent power of civil rights laws as well as agency and private enforcement actions.^73^

Adjudicating legal rights, however, may be more costly and less effective than ex ante interventions precisely because of lost time, money, the inability to capture the entirety of the harms (including emotional and stigmatic harms), and the predominance of individual rights rather than collective actions at the heart of private enforcement actions.^74^ It also concerns access to courts or agencies which require time, money, and in some cases, legal counsel. This situates lawyers and legal experts (many of whom are medical professionals) as the drivers of the remedial process who shape the narratives of harm and imagine available remedies. However, both disability and health justice seek to redistribute power to those most marginalized and affected by social injustice. Reliance on purely legal remedies remains limited and may run counter to this explicit goal.

## Conclusion

Disability is an important independent variable in the health justice framework. Incorporation of disability as demographic into a health justice framework does not require abandonment of disability as a measure of health inequities. In fact, the two perspectives, as this article, Aida’s case study, and the work of Professor Rabia Belt have shown, are critical for advancing both disability and health justice. As Professor Belt aptly describes “Disability is the connecting point between the body and society, where social injustice becomes material. Thus, a discussion about fatness in prison and slow violence is not just about current doctrinal outcomes; it is also about the gradual whittling away of resources in poor and Black and brown communities, the rise of mass incarceration, and the shifting of resources from the welfare state to the carceral state.^75^ What does it mean to incorporate disability in this way? In addition to the examples provided in this article, health justice researchers and practitioners should push for disability as a standardized variable in official data collections and research studies.^76^ Beyond its expressive value, collection of disability data will further document the experiences of people like Aida and her family as they seek redress of institutional harms. Disability data paired with preexisting data on the social determinants such as housing (typically of interest to and reviewed by health justice scholars) can strengthen claims of environmental injustice (formally through the legal system and, perhaps more importantly, informally as a matter of community organizing and policy advocacy). But the fact that the un-remediated housing conditions may have contributed to (or even caused) Aida’s and Marco’s disabilities does not mean that they will not or do not see themselves as disabled. Aida may identify as disabled for a number of reasons ranging from access to SSI to her own identity as a disabled Latina for purposes of political mobilization or personal relationships. Such identity construction remains complex and fraught with contradictions and trauma.

The choice of how to identify belongs to Aida yet disability is an important variable in research and practice even if Aida personally chooses not to identify as disabled. Theoretically, Aida’s bodily and neurological non-normativity place her outside of society’s conjured personification of physical strength, exacting rationality, and unrelenting fiscal independence that fuels the American political economy, what Professor Rosemarie Garland-Thomson has called the “normate.”^77^ Locating disability in Aida’s case identifies the structural barriers contributing to Aida’s health inequities, the “slow violence” experienced by Aida because of environmental harms, legal constructions of disability that keep Aida and her family below the poverty line, and laws that regulate the decisions of Aida and her family precisely because legal institutions rely on assumptions of decisional incapacity as the norm for certain disabled individuals. Practically, Aida is part of an unjust ecosystem and health justice requires critical situatedness to understand intersectional harms and meaningfully address them. Disability and health justice are not diametrically opposed but complex and mutually constitutive. In this sense, a health justice framework is one that recognizes the difficultly of locating disability and rejects the one-dimensional draw of reducing disability to a data point upstream (demographic) or downstream (health outcome). Instead, deploying disability as an analytical lens may avoid limiting the questions asked in research or practice or the remedies sought that assume disability is simply the result of discrimination and injustice for people with other marginalized identity axes. For example, equitable employment as a marker of health justice should avoid reinforcing conceptions of economic productivity that may leave some disabled people out by privileging those who can meet existing expectations of full employment. Thus, using full time employment as a marker of health justice for Aida may miss the fact that Aida’s disabilities do not allow her to work the standard forty-hour week. The Social Security Act’s SSI provisions certainly narrow her choices to ensure access to health insurance and continued receipt of the monthly stipend, but the solution is not to get her access to a forty-hour work week and private health insurance. Instead, scholars and practitioners attending to disability must wrestle with the institutional designs tethering private employment and health insurance and imagine what justice would look like for someone who may never work a typical full-time job because she simply cannot. What does health justice look like outside of the current political economy? This article (and the symposium it reflects) invites these difficult questions in service of health justice.”
